# Therapeutic effects of clonazepam in patients with burning mouth syndrome and various symptoms or psychological conditions

**DOI:** 10.1038/s41598-023-33983-6

**Published:** 2023-05-04

**Authors:** Hyun-Il Shin, Joo-In Bang, Geun-Jeon Kim, Mi Ra Kim, Dong-Il Sun, Sang-Yeon Kim

**Affiliations:** 1 Department of Otorhinolaryngology-Head and Neck Surgery, College of Medicine, The Catholic University of Korea, 222 Banpo-daero, Seocho-gu, Seoul, Korea; 2 Department of Otorhinolaryngology-Head and Neck Surgery, College of Medicine, The Inje University of Korea, Busan, Korea

**Keywords:** Cancer, Oncology

## Abstract

Burning mouth syndrome (BMS) is frequently accompanied by dysgeusia and xerostomia. Clonazepam has been widely prescribed and is effective, but it is unclear whether clonazepam also affects the symptoms that accompany BMS, or whether such symptoms affect treatment outcomes. Here, we investigated the therapeutic outcomes in BMS patients with various symptoms or comorbidities. We retrospectively reviewed 41 patients diagnosed with BMS between June 2010 and June 2021 at a single institution. Patients were instructed to take clonazepam for 6 weeks. Before the first dose, burning pain intensity was measured using a visual analog scale (VAS); the unstimulated salivary flow rate (USFR), psychologic characteristics, site(s) of pain, and any taste disturbance were evaluated. Burning pain intensity was measured again after 6 weeks. Thirty-one of the 41 patents (75.7%) exhibited a depressed mood, whereas more than 67.8% of the patients exhibited anxiety. Subjective xerostomia was reported by ten patients (24.3%). The mean salivary flow rate was 0.69 mL/min and hyposalivation (an unstimulated salivary flow rate ≤ 0.5 mL/min) was apparent in ten patients (24.3%). Dysgeusia was present in 20 patients (48.7%); a bitter taste (n = 15, 75%) was reported by the largest proportion of patients. Patients who reported a bitter taste responded best in terms of burning pain reduction after 6 weeks (n = 4, 26.6%). Overall, 32 patients (78%) reported decreased oral burning pain after clonazepam (mean VAS score changed from 6.56 to 5.34) use. Patients who reported taste disturbances exhibited a significantly greater decrease in burning pain, compared with other patients (mean VAS score changed from 6.41 to 4.58) (*p* = 0.02). Clonazepam significantly improved burning pain in BMS patients who had taste disturbances.

## Introduction

Burning mouth syndrome (BMS) is a complex disorder, usually present in older women (1.5–5.5% of older women), and is characterized by pain and a burning sensation in the mouth but no visible mucosal abnormality^1^. The principal symptom is pain, often accompanied by oral dryness (xerostomia) and dysgeusia. Further, BMS is often associated with psychological issues (depression or anxiety)^2,3^, hormonal changes, local effects (lichen planus or candidiasis), and systemic dysfunctions. Although many authors have studied BMS pathogenesis, the etiology remains unclear but is probably multifactorial, involving complex interactions between local, systemic, and/or psychogenic factors. BMS has been divided into two types: spontaneous (or primary) BMS, and Secondary BMS may be caused by complex interactions between local factors (e.g., hyposalivation) or systemic/psychogenic factors, but a clear definition of secondary BMS remains elusive^4–6^. Based on recent findings, primary BMS is now believed to be a form of neuropathic pain^7,8^. With respect to secondary BMS, there are findings for local or systemic factors^1,9–11^. Among them, many studies have focused on psychologic issues, hyposalivation, and disturbance of the taste sensory pathway as causes of BMS^1,12,13^. Depressive mood (in up to 35% of patients) or anxiety trait (in up to 50% of patients) have been reported frequently in BMS patients^1,13,14^. In addition, approximately 25% of BMS patients exhibit xerostomia, and two-thirds of patients report taste disturbances^1,11^. However, the exact cause has not been proven to date^1,6^. Thus, diagnosis and management remain challenging, and the responses to various treatments have been inconsistent and limited. In the meantime, several studies have been conducted on the treatment of BMS; representative examples include clonazepam therapy, which has demonstrated relatively consistent therapeutic effects^15–18^. However, compared to the frequent accompaniment of xerostomia and taste change or psychogenic factors in BMS patients, studies on the therapeutic effect of clonazepam in the presence of these medical conditions or comorbidities have not been well studied. Here, we investigated the effects of clonazepam on xerostomia, taste disturbances, dysgeusia, and psychogenic conditions.

## Methods

### Patients and study design

From June 2020 to June 2021, we retrospectively enrolled consecutive patients with BMS. This study included 41 patients who visited our otorhinolaryngology outpatient department for treatment of intraoral burning or dysesthesia and were then diagnosed with BMS. The inclusion criteria were established in accordance with the 2013 International Classification of Headache: intraoral burning or dysesthesia daily for > 2 h/day for > 3 months, without any clinically evident causative lesion. Pain has both of the following characteristics: (1) burning quality, (2) felt superficially in the oral mucosa^19^. The exclusion criteria were: current usage of benzodiazepines; allergy to benzodiazepines; and any serious disease of the central nervous system. At the first visit, an oral examination, Beck Depression Inventory (BDI), State-Trait Anxiety Inventory (STAI) questionnaire, and the comprehensive questionnaire were provided to the patients. Patients were then interviewed by one doctor and received an explanation regarding the possible etiology and management strategies for BMS. At the second visit, scheduled in the morning, the salivary flow rate was measured, the BDI and STAI was recollected, and the comprehensive questionnaire was checked by the staff to ensure the completion of any omitted sections. Then, patients were instructed to take 0.75 mg clonazepam (one half tablet) three times daily for the first 2 weeks (2.25 mg daily). If no severe drowsiness and/or dizziness developed, the dose was increased to 1.5 mg (one full tablet) three times daily for the remaining 4 weeks (4.5 mg daily). Patients were instructed to place clonazepam tablets sublingually, allow them to dissolve, and then swallow. Questionnaires were completed before treatment and after 6 weeks of treatment. All patients completed treatment without side effects.

This study was carried out in accordance with the ethical standards laid down in the 1964 Declaration of Helsinki and its later amendments. All study procedures were approved by the Institutional Review Board of the Inje university (IRB No. 2022-08-005-001). All the participants gave their informed written consent and all methods were performed in accordance with the relevant guidelines and regulations.

### Questionnaire

The questionnaire used to evaluate subjective symptoms included questions about duration of suffering, area of symptoms, type of discomfort. Burning sensation was assessed using a visual analog scale (VAS) that ranged from 0 to 10, with 0 indicating no pain and 10 indicating the worst possible pain. Additionally, the following items were explored in the questionnaire. Before treatment items were pain location; time of day when pain was most severe; pain duration; factors affecting pain (eating, type of food, talking, any drug); any change in taste (and the extent of such a change); dry mouth intensity; time since onset of burning; and whether dry mouth was worst before or after burning symptom onset. After treatment (6 weeks) items were burning sensation improvement (VAS score); region of improvement; and any change in taste.

### Salivary flow rate

Saliva was collected by a standardized method as described elsewhere^20,21^. We measured unstimulated salivary flow rates (uSFRs) in patients at rest in a quiet room. Samples from the subjects were collected between 9:00 and 11:00 a.m., to minimize diurnal variability. All subjects abstained from smoking, eating, and drinking for 2 h prior to the measurement of salivary flow rate. The collection of unstimulated saliva started with the instruction to void the mouth of saliva by swallowing. Subsequently, saliva was allowed to ac cumulate in the floor of the mouth, without stimulation of saliva secretion by means of orofacial movements. After 5 min, participants were told to expectorate residual saliva into the container and unstimulated saliva flow rates were read. Then, patients were asked to collect saliva and spit it into a test tube for 5 min. The flow rate of whole saliva was expressed as mL/min. Hyposalivation was defined as a uSFR ≤ 0.1 mL/min^22^.

### Psychologic status

We used the Beck Depression Inventory (BDI) and State-Trait Anxiety Inventory (STAI). Beck Depression Inventory (BDI) is a self-reported instrument that measures attitudes and symptoms characteristic of depression^23^. The 21-item BDI measures the extent of depression (0–9, no depression; 10–18, mild-to-moderate depression; 19–29, moderate-to-severe depression; and 30–63, severe depression). State-Trait Anxiety Inventory (STAI) actually classifies patients who have anxiety as a trait, i.e. personality characteristic while anxiety as a state reflects current anxious state of the person. STAI questionnaire is used to diagnose and measure state and trait anxiety and also to distinguish it from depressive syndromes^24^. The STAI includes two 20-item subscales: the State Anxiety Scale (S-Anxiety) evaluates state anxiety (i.e., how a respondent feels “right now”), whereas the Trait Anxiety Scale (T-Anxiety) evaluates longer-term “anxiety proneness”. A score of 20–37 indicates no or low anxiety, while scores of 38–44 and 45–80 indicate moderate anxiety and high anxiety, respectively.

### Statistical analysis

Data were compared using the chi-square, Fisher’s exact, paired-t, or Wilcoxon-signed rank test, as appropriate. In comparing demographic and clinical data, the authors used Student’s t-test for continuous data and Chi-square tests or Fisher’s exact tests for discrete data. All calculations were performed using IBM SPSS Statistics for Windows ver. 25 (IBM Corporation, Armonk, NY, USA). The level of statistical significance was set to P < 0.05.

## Results

### Patient characteristics

Forty-one patients (four men and 37 women) were included. Table 1 lists the descriptive and demographic characteristics of the patients. The mean age was 66.5 ± 7.92 years; two patients (4.8%) had a history of diabetes, 23 patients (56%) had a history of hypertension, and seven patients (17%) had a history of psychologic treatment. Subjective burning severity was high (VAS score of 6–10) in 65.8% of patients; the mean VAS score was 6.56 ± 1.92. Pain was most commonly worst in the afternoon (58.5%), followed by the night (53.6%), immediately after waking (36.5%), and the morning (36.5%). All patients reported burning pain in the tongue (n = 41, 100%) followed by the buccal mucosa (n = 12, 29.2%) and palate (n = 12, 29.2%); 24 patients (58.5%) reported pain in > 2 sub-sites. The mean dry mouth VAS score was 5.48 ± 3.0 and the mean USFR was 3.45 ± 4.43 mL/min. Hyposalivation was observed in ten patients (24.3%); all also reported xerostomia. Twenty patients reported dysgeusia (24.3%) (Table 1).

**Table 1:** Clinical characteristics of patients.

Characteristics	N = 41
Age [years, mean (SD)]	66.5 ± 7.92
Sex (M:F)	4:37
Comorbidities	
DM	2 (4.8%)
HTN	23 (56%)
History of psychologic treatment	7 (17%)
Mean BMS VAS [mean (SD)]	6.56 (1.92)
Time since onset	
Less than 1 month	5 (12.1%)
1 to 6 months	19 (46.3%)
6 to 12 months	3 (7.3%)
More than 1 year	14 (34.1%)
The worst time	
Immediately after waking up	15 (36.5%)
Morning	15 (36.5%)
Afternoon	24 (58.5%)
Nighttime	22 (53.6%)
Involved subsite*	
Tongue	41 (100%)
Palate	12 (29.2%)
Lip	9 (21.9%)
Buccal mucosa	12 (29.2%)
Gingival mucosa	6 (14.6%)
Mouth floor	8 (19.5%)
Tooth	3 (7.3%)
Dysguesia	20 (48.7%)
Hyposalivation (salivary flow rate ≤ 0.5 ml/min)	10 (24.3%)
Mean dry mouth VAS [mean (SD)]	5.48 (3.0)
Mean salivary flow rate [mean (SD), mL/5 min]	3.45 (4.43)

*Total sum exceeds 100% due to duplicity.

### Psychological status

The BDI showed that 19 patients (46.3%) had mild-to-moderate depression, six patients (14.6%) had moderate-to-severe depression, and six patients (14.6%) had severe depression. The STAI-S showed that 12 patients (29.2%) exhibited moderate state anxiety and 18 patients (43.9%) exhibited severe anxiety. The STAI-T showed that 12 patients (29.2%) exhibited moderate trait anxiety and 18 patients (43.9%) exhibited severe anxiety (Table 2).

**Table 2:** Psychological status of study participants.

Characteristics	N = 41
BDI characteristics	
No depression	10 (24.3%)
Mild to moderate depression	19 (46.3%)
Moderate to severe depression	6 (14.6%)
Severe depression	6 (14.6%)
STAI-S characteristics	
No or mild anxiety	14 (34.1%)
Moderate anxiety	13 (31.7%)
Severe anxiety	14 (34.1%)
STAI-T characteristics	
No or mild anxiety	11 (26.8%)
Moderate anxiety	12 (29.2%)
Severe anxiety	18 (43.9%)

*BDI* Beck Depression Inventory, *STAI* State-Trait Anxiety Inventory.

### Burning pain improvement according to sub-site

**Figure 1:**
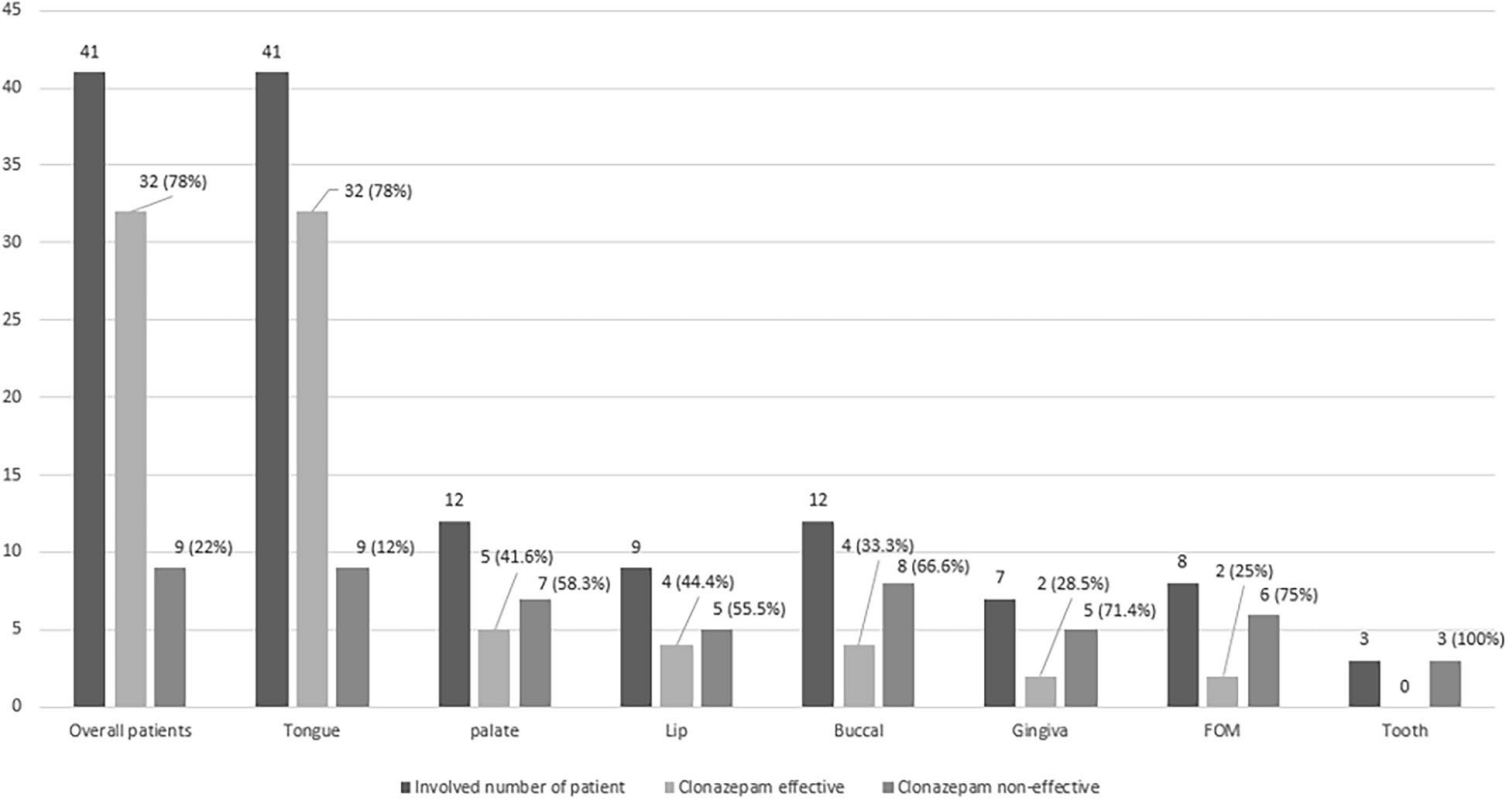
Effect of clonazepam according to sub-site.

Of the 41 patients, 32 (78%) reported that clonazepam was effective (mean VAS score changed from 6.56 to 5.34). The changes in pain severity in the tongue, palate, lip, buccal area, gingiva, mouth floor, and teeth are listed in Table 3. The summed incidences exceed 100% because of duplications. Clonazepam efficacy did not significantly differ according to the number of affected sites. The most effective response was observed in the tongue (n = 32, 78%), followed by the lips (n = 4, 44.4%) and palate (n = 5, 41.6%) (Fig. 1).

**Table 3:** Burning pain change effect of clonazepam according to sub-site.

Tongue (41)		Palate (12)		Lip (9)		Buccal (12)		Gingiva (7)		FOM (8)		Tooth (3)	
+	−	+	−	+	−	+	−	+	−	+	−	+	−
32	9	5	7	4	5	4	8	2	5	2	6	0	3

	Effective	Non-effective	p-value										
No. of involved site													
1 site (n = 17)	15 (88.2%)	2 (11.7%)	0.25										
2 sites (n = 10)	6 (60%)	4 (40%)											
≥ 3 sites (n = 14)	11 (78.5%)	3 (21.4%)

### Effect of therapy according to psychological status

Patients were divided into subgroups according to the BDI, STAI-T, and STAI-S scores (Table 4). Nine patients (90%) responded (mean VAS score changed from 6.32 to 5.25) in the no-depression group (n = 10), whereas 23 patients (74.2%) responded in the depressive mood group (mean VAS score changed from 7.3 to 5.6). According to STAI status, 11 of 14 patients (78.5%) with no or mild anxiety responded (mean VAS score changed from 6.33 to 5.44), whereas 21 of 27 patients (77.7%) with moderate or severe anxiety responded (mean VAS score changed from 7 to 5.14) (Fig. 3). However, the differences were not statistically significant.

**Table 4:** Clonazepam effect according to psychologic state.

Subgroup	BMS VAS effect		
	Improved	Non-effective	p-value
BDI			
No depression (n = 10)	9 (90%)	1 (10%)	0.41
Mild to severe depression (n = 31)	23 (74.2%)	8 (25.8%)	
STAI-S			
No or mild anxiety (n = 14)	11 (78.5%)	3 (21.4%)	0.64
Moderate or severe anxiety (n = 27)	21 (77.7%)	6 (22.2%)	
STAI-T			
No or mild anxiety (n = 11)	9 (81.8%)	2 (18.1%)	0.54
Moderate or severe anxiety (n = 30)	23 (76.6%)	7 (23.3%)	

*BDI* Beck Depression Inventory, *STAI* State-Trait Anxiety Inventory.

### Effect of therapy according to salivary flow rate

Patients were divided into subgroups according to the USFR (Table 5). In the hyposalivation group (n = 10), seven patients (70%) responded (mean VAS score changed from 7.6 to 5.9). In the normal salivation group (n = 31), 25 patients (80.6%) responded (mean VAS score changed from 6.2 to 5.16). The difference was not statistically significant.

**Table 5:** Clonazepam effect according to salivary flow rate.

Subgroup	BMS VAS effect		
	Improved	Non-effective	p-value
Hyposalivation (n = 10) (salivary flow rate ≤ 0.5 ml/min)	7 (70%)	3 (30%)	0.38
Normal salivation (n = 31) (salivary flow rate > 0.5 ml/min)	25 (80.6%)	6 (19.3%)	

### Effects of therapy according to dysgeusia status

**Figure 2:**
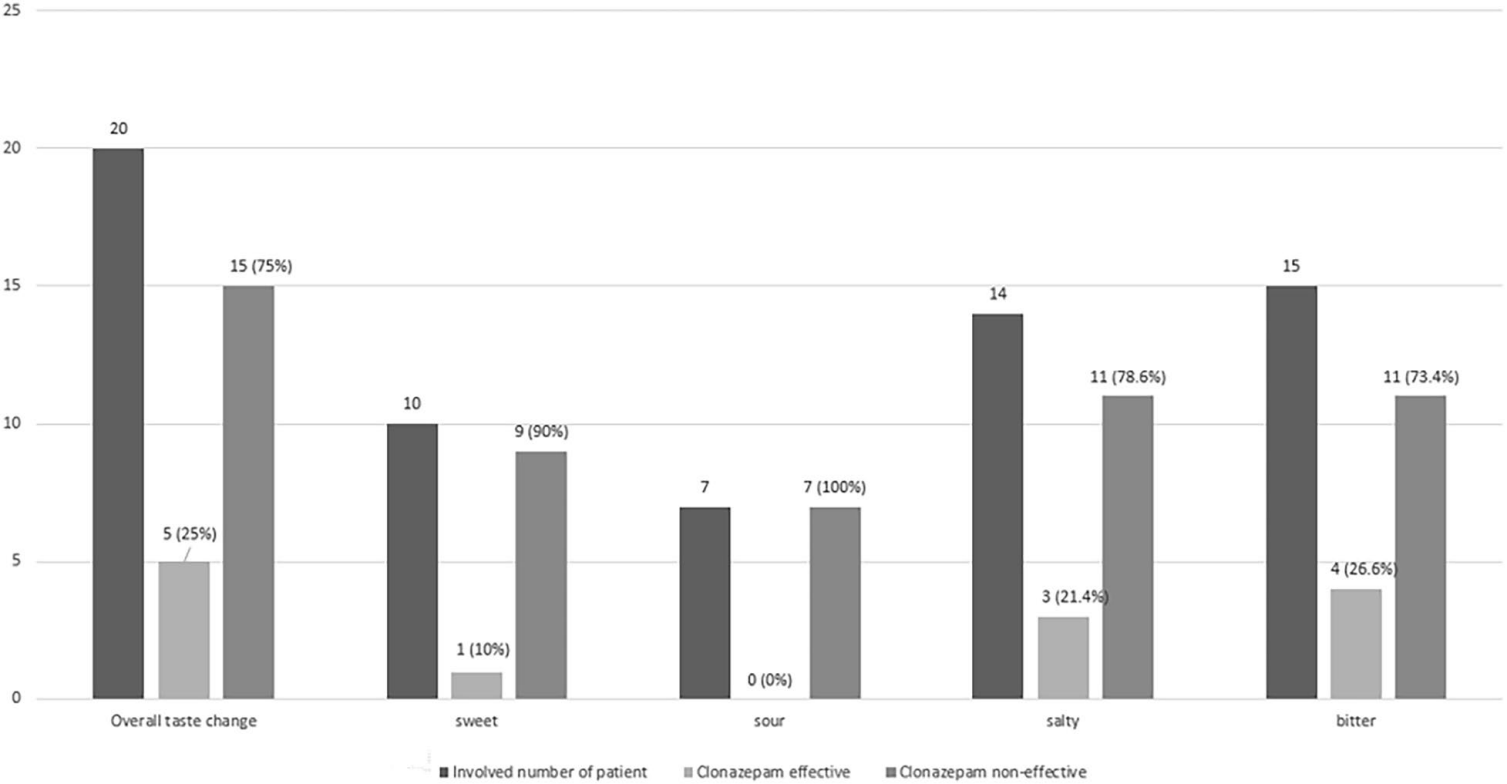
Effect of clonazepam according to altered taste.

**Figure 3:**
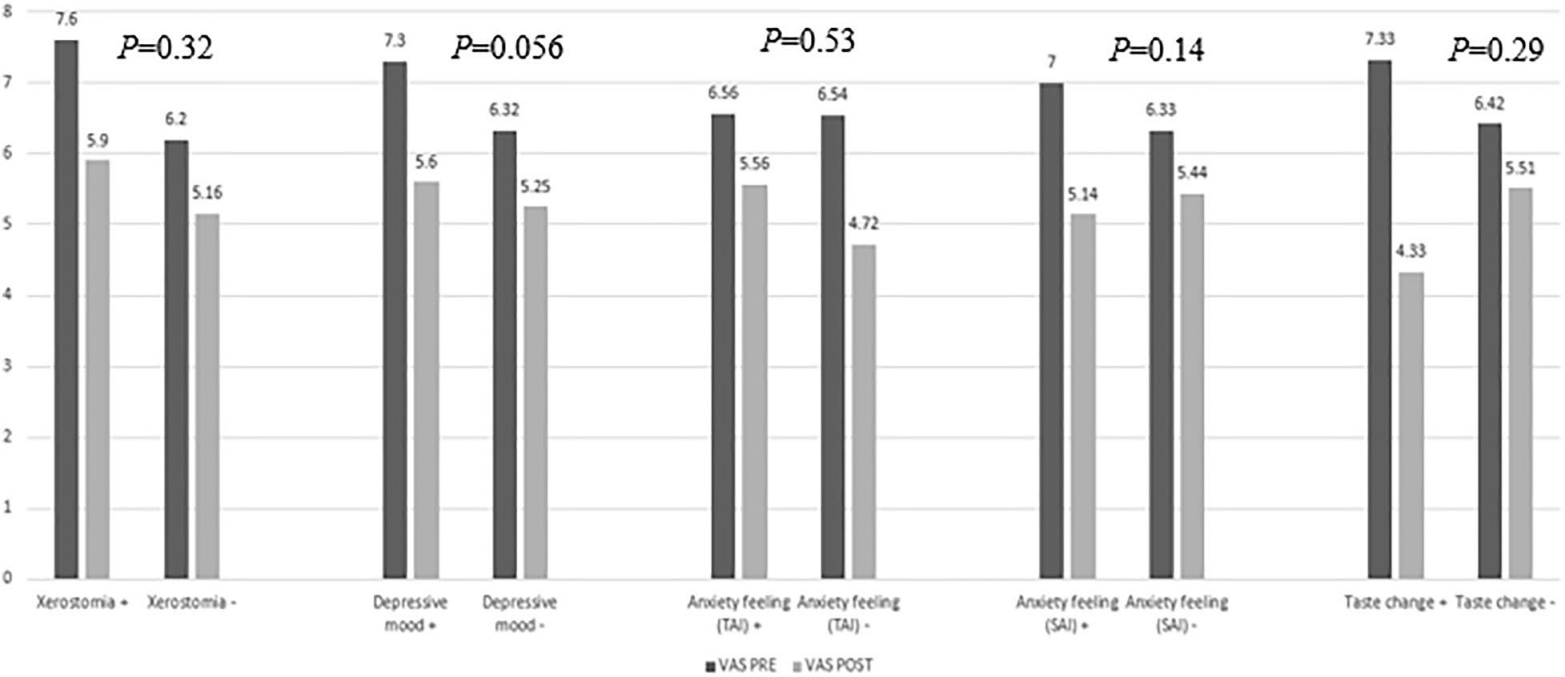
BMS VAS change after clonazepam use according to the accompanying symptoms.

The effects of clonazepam on taste are listed in Table 6. Twenty patients (48.7%) reported taste disturbances, whereas five patients (25%) reported improvement after treatment (mean VAS score changed from 7.33 to 4.33) (Figs. 2, 3). The summed incidences of taste types exceed 100% because of duplication. Patients who reported bitter taste responded best (in terms of burning pain reduction) (n = 4, 26.6%), followed by patients who reported salty taste (n = 3, 21.4%) (Fig. 2). The difference was not statistically significant.

**Table 6:** Improvement of dysgeusia after administration of clonazepam.

Overall change (20)		Sweet (10)		Sour (7)		Salty (14)		Bitter (15)	
+	−	+	−	+	−	+	−	+	−
5 (25%)	15 (75%)	1 (10%)	9 (90%)	0	7 (100%)	3 (21.4%)	11 (78.6%)	4 (26.6%)	11 (73.4%)

+ (effective), − (non-effective).

### Burning pain improvement according to combinations of accompanying symptoms

We analyzed three subgroups: burning pain only (n = 9), burning pain with taste disturbance (n = 17), and burning pain with xerostomia (n = 10). Patients who reported concurrent burning pain, taste disturbance, and xerostomia (n = 5) were excluded. After treatment, all three subgroups responded, but only the second subgroup reported significant improvement in burning pain (mean VAS score changed from 6.41 to 4.58, *p* = 0.02) (Table 7).

**Table 7:** Changes in the mean VAS score by clonazepam use according to the accompanying oral complaints.

Subgroup	N	Baseline		6 weeks		P value
		Mean	SD	Mean	SD	
Only burning	9	6.0	1.96	5.85	2.44	0.84
Burning & taste disturbance	17	6.41	1.58	4.58	2.64	0.02
Burning & xerostomia	10	7.6	2.17	5.9	3.21	0.25

## Discussion

Due to its complex nature, the definition of BMS has been changed over time. A recent international study using the Delphi method found that experts are of the view that the ICD-11 term, disease description, and diagnostic criteria for BMS should be revised and the condition termed burning mouth disorder (BMD)^25^. BMS has been divided into two types, thus primary (there is no other disease) and secondary (possibly attributable to a local or systemic disorder)^11^. Primary BMS is idiopathic, in that the organic local/systemic causes cannot be identified; however, peripheral and central neuropathological pathways are potentially involved^9^. Secondary BMS may feature a local or systemic condition such as a mucosal disease (i.e., lichen planus or candidiasis); a hormonal disturbance; a psychosocial stressor; a vitamin or nutritional deficiency; diabetes; dry mouth; a contact allergy; a parafunctional habit; a cranial nerve injury; or a side-effect of medication, but this remains unclear^1,11,26^. Primary BMS treatments seek to alleviate symptoms; secondary BMS requires diagnosis and treatment of the underlying condition^11^. There were substantial differences in burning symptom cessation with treatment; the patients who had secondary BMS improved if the underlying clinical abnormality was treated, whereas the primary BMS group rarely reported such positive results^11^. Meanwhile, the main accompanying medical conditions or comorbidities of BMS are xerostomia, dysgeusia, or psychological issues such as depression and anxiety^2,3^. Thus, clinicians have attempted to manage BMS along with xerostomia, taste disturbance, and psychogenic issues. In general, clonazepam has been recognized as the most effective standard treatment, regardless of the accompanying symptoms or type of BMS^15–18^; its therapeutic effect is mediated by the inhibition of pain signaling^16^. Clonazepam is known as an agent for the inhibitory neurotransmitter gamma-amino butyric acid (GABA), and GABA receptors are distributed in the oral pain and taste signaling pathways^12,27^. Therefore, clonazepam may have the effect of reducing burning pain in the oral cavity.

Optimal administration route for clonazepam in BMS patients has been explored by many studies. A few studies on topical clonazepam demonstrated both significantly decreased pain scores and improvement of pain/burning symptoms in BMS patients^28–30^. Rodriguez et al. gave detailed data and analysis of all 66 patients, including a gradual increase in number of pills used in the placebo group compared to that of the treatment group, and affirmed the benefit of the medication^29^. Some other studies investigated the systemic effect of clonazepam^18,31^. Heckmann et al. demonstrated ingested clonazepam showed a significant decrease in pain scores, but reanalysis of the data failed to show a significant change between the initial and final stages of the therapy^18^. On the other hand, Grushka’s study presented a consistent pain reduction in systemic clonazepam treatment^31^. To see topical as well as systemic effects, Amos et al. explored combined administration methods^32^. This study found that intraoral clonazepam was superior to oral ingestion; pain was much more rapidly alleviated but the duration of action was reduced^32^. As such, there have been several studies on the effect of clonazepam according to the route of administration, but there is no consensus as to which is the optimal method, with a large-scale study yet to take place. Under this circumstance, clonazepam tablets were placed sublingually to see both local and systemic effects in this study.

BMS is often accompanied by complaints of xerostomia, as reported by 25–40% of BMS patients in recent studies^1,11,33^. Scala et al. reported that 46–67% of BMS patients claim to be experiencing xerostomia, regardless of whether the salivary flow rate is decreased; notably, dry mouth is often subjective, as opposed to any real decrease in salivary gland function^9,26^. However, the response to clonazepam treatment for BMS in those with xerostomia is not clear, as only a few studies have examined this. In one of them, Silva reported that the therapeutic response of topical urea treatment (10% of concentration, applied to the oral cavity three to four times per day for 3 months) was 60% in patients with xerostomia, which was similar to the results obtained with the control group^34^. In our present work, ten patients (24.3%) out of 41 reported subjective xerostomia, and all ten of these patients exhibited objective hyposalivation. Among the hyposalivation patients, seven out of ten (17%) responded to clonazepam in terms of a reduced BMS visual analogue score (VAS) score (mean VAS changed from 7.6 to 5.9); however, the response rate was lower than that (80%) of the normal group, which was not statistically significant. Previous studies have presented evidence suggesting that changes in the salivary flow rate may be associated with mucosal atrophy and/or subclinical inflammation, which may be accompanied by oral neuropathy in BMS patients^34–36^. Lauria et al. also demonstrated oral neuropathy by examining the tongue epithelium of BMS patients and found fewer small-diameter nerve fibers, which may explain the thermal hypoesthesia symptoms and change in the pain thresholds. Considering the above, it is thought that dry mouth is closely related to BMS onset. In addition, it can be hypothesized that the effect of clonazepam may be reduced as chronic neural degeneration occurs extensively as the duration of dry mouth is extended. Therefore, we think that this study can provide some insight into predicting the treatment effects of BMS patients with xerostomia.

From the aspect of psychogenic factors, several studies have suggested that psychological factors cause BMS^1,11,13,14,37–39^. Depressive mood, anxiety, somatization, and aberrant personality traits have frequently been reported in BMS patients, and much of the research has been devoted to the role of psychological factors in BMS^1,13,14,38^, in which psychosocial disorders with a principal focus on anxiety and/or depression were examined. Several studies reported that up to 35% of BMS patients showed depressive mood and up to 50% of patients complained of anxiety^1,14,38^. On the other hand, some controversy remains as to whether psychogenic pathological conditions occur primarily or are secondary to BMS. In general, psychological disorders may be associated with the modulation of pain perception, increasing nerve transmission by peripheral pain receptors, and altering of an individual’s perception of pain. Based on this, with the belief that the treatment effect for each BMS patient may differ depending on the accompanying psychogenic problem, some studies have shown that psychological problems are associated with worse prognoses^9,26,40^. According to Ko's study, the reduction in burning pain caused by clonazepam was smaller in the psychologic disease group than the control group (29% vs. 45%, respectively)^26^. Among our patients, depressive mood and anxiety trait were reported by 75.7% and 65.9%, respectively. Like other studies, our study found that the response rate with clonazepam therapy on burning pain was less in patients with psychologic problems than in those without (in depressive mood: 74.2% vs. 90%; in anxiety trait: 77.7% vs. 78.5%, respectively). However, there was no significant improvement in the BMS VAS score, regardless of the Beck Depression Inventory or State Trait Anxiety Inventory (T/S) score. The small degree of pain reduction in patients with psychogenic problems may be explained by emotional and cognitive factors for chronic pain. Forssell et al. demonstrated that BMS patients with more intensive and interfering pain report more depressive and pain-related anxiety symptoms, compared to patients with less severe pain intensity^41^; he explained this using a biopsychosocial model of chronic pain, showing that emotional and cognitive factors play a role in pain experience. Forssell also claimed that BMS patients with more intensive pain reported more depressive- and pain-related anxiety symptoms, and more hypervigilance to pain, compared to patients with less severe pain intensity^41^. Excessive attention to pain or pain hypervigilance has been reported in association with a higher pain intensity, disability, and emotional distress in different pain patient populations^42^. Such patients usually exaggerate their pain, thus they are likely to express a poor response to clonazepam^26^. Also, patients with psychogenic symptoms are likely to take psychologic medications. According to Ko's study, 23% of BMS patients had been taking psychologic medications; these patients showed significantly reduced salivary flow rates because psychologic medications may decrease salivary gland function and may result in a reduction of saliva’s protective capacity in the mouth^26^. As described above, the treatment effect of clonazepam may be influenced by a change in salivary components and xerostomia induced by psychologic medication.

Especially, menopause has been suggested to be a major cause of BMS. Earlier reports found that BMS affected primarily postmenopausal women^9,13^, and suggested that changes in the female sex hormone levels may predispose women to BMS. As both BMS and vulvodynia are far more common in postmenopausal than menstruating women, estrogen deficiency may be a shared etiological mechanism; estrogen receptors have been identified in the tongue, salivary gland, and vaginal mucosae^43^. Some reports stated that hormone replacement therapy in postmenopausal patients with oral discomfort was useful^44^; however, more research is needed.

A loss of taste inhibition by central structures that mediate oral pain has been proposed to explain BMS development^13^. Repetitive nociceptive inputs against a background of peripheral neuropathy eventually elicit central sensitization and other changes. Grushka proposed that BMS reflects the persistent breakdown of an intrinsic equilibrium caused by a reduction in corda tympani function; subsequently, lingual nerve inhibition and central sensitization tend to occur^1^. Multiple studies have revealed damage to the innervation areas of the corda tympani and glossopharyngeal nerves in BMS patients; these areas control bitter taste. Such selective inhibition may reflect the loss of central pain inhibition^1,27,45^. Among our patients with taste disturbances, most of them showed changes in bitter taste; these patients responded best to treatment, supporting the selective inhibition hypothesis described above. Although the role of taste in terms of oral burning syndrome is complex, clonazepam is an agonist of the inhibitory neurotransmitter GABA, which is active in the oral mucosa, mandible, palate, and salivary gland. Above all, it can be noted that it also acts on the taste pathway^12^. If burning causes taste disturbances that result in the loss of normal inhibition of a central structure that mediates oral pain, GABA-specific agents may relieve such pain. We found that the pain VAS scores improved significantly after treatment in patients with taste disturbances. Based on the contents described above, we assume that when taste change accompanies the treatment response, there is a better effect on pain improvement.

There have been many studies reporting the prevalence of psychogenic conditions, and taste disturbance in patients with BMS^26,29^. However, studies on the therapeutic effect of clonazepam in the presence of these concomitant symptoms or comorbidities have been insufficient. Thus, we assessed the therapeutic effects according to psychological, dysgeusia, and hyposalivation status. We found no correlation of psychological or hyposalivation status with the therapeutic effect of clonazepam; however, taste disturbance was associated with a higher response rate and greater improvement in relieving the burning pain. Our work had several limitations. First, it was questionnaire-based and retrospective. Second, the sample size was small. Third, it was a single-center study. Finally, we could not confirm that clonazepam had been appropriately taken; nevertheless, we found that clonazepam significantly improved the burning sensations of BMS patients with taste disturbances. Such information can be employed during patient counseling and to predict therapeutic outcomes.

## Conclusion

A large proportion of BMS patients also experience xerostomia, psychogenic symptoms, and taste change. Clonazepam significantly reduced burning pain in BMS patients with taste disturbances, but no significant reduction in burning pain was found in BMS patients with xerostomia or psychogenic traits only. Such information can be used in BMS patient counseling and the prediction of therapeutic outcomes with clonazepam treatment.

## Data Availability

The datasets used and/or analyzed during the current study available from the corresponding author on reasonable request.
